# Our Second Volume

**Published:** 1880-11

**Authors:** 


Our Second Volume.—The December issue will close the first volume of *The Independent Practitioner.* We have striven to make, and to keep it as nearly up to the principles announced in its *prospectus* as possible; and have the gratification of knowing that it has met the hearty endorsement and commendation of many of the wisest and best members of the profession to whom it pays its monthly greetings. While it continues to be thus acceptable to the enlightened and liberal leaders of thought and supporters of the profession, its course must be onward and upward in the career on which it is embarked. That it would not prove acceptable to *all* who might peruse its pages, was confidently expected ere its advent, and in fact, any journal standing upon the high ground it occupies, will always find some among the lesser lights whom it cannot please, and especially among the vulnerable ones who have but recently emerged from their primordial obscurity, and who naturally "despise the excellence they cannot reach" at a single bound. But even these should know, that "acting well their part," may in time give them status and consideration, which can never be reached by captious refusal of the experience of more thoughtful and liberal minds. We intend to keep it such a journal as intelligent and honorable members of the profession would like it to be. To this end, we respectfully and earnestly invite contributions and subscriptions *from every quarter.* If our friends will promptly respond to this appeal, the second volume shall be even a marked improvement upon the present one. And with assurances of profound gratitude to *all those* whose encouragement and favor we have received, we shall press steadily forward toward the goal for which our labors were begun!

